# A Delphi consensus study to determine the workload components and activity standards of dietitians in South Africa’s central and tertiary public hospitals

**DOI:** 10.1186/s12960-023-00883-9

**Published:** 2024-01-08

**Authors:** Vertharani Nolene Naicker, Keshan Naidoo, Jane W. Muchiri, Modiehi Heather Legodi

**Affiliations:** 1 National Department of Health, Dr AB Xuma Building, 112 Voortrekker Road, Pretoria Townlands 351-JR, Pretoria, 0187 South Africa; 2 Right to Care, 1006 Lenchen Avenue North, Centurion, Pretoria, South Africa; 3 Department of Human Nutrition, Faculty of Health Sciences, University of Pretoria, Pretoria, South Africa

**Keywords:** Activity standards, Delphi, Dietitian, Hospitals, South Africa, Staffing need, Workload components, Workload indicators

## Abstract

**Background:**

The global Human Resources for Health (HRH) strategy emphasizes the need to invest in HRH to meet population needs and improve the provision of quality health care services. In South Africa, dietitians are recognized as registered professionals who provide nutrition services. In this paper, we used 2 key steps (3 and 4) of the eight step World Health Organization (WHO) Workload Indicators of Staffing Need (WISN) methodology to determine the workload components and activity standards for dietitians at South African central and tertiary public hospitals.

**Methods:**

All (9) provincial nutrition managers (phase one) and 21 out of a total 22 head dietitians at central and tertiary public hospitals (phase two) participated in an online survey. In phase one, the provincial managers provided the job descriptions (JDs) of dietitians in their provinces, and the JDs were analyzed to determine the baseline workload components. In phase two, dietitians participated in a multi-stage Delphi process to reach consensus on workload components and activity standards. Consensus was deemed to be agreement of 70% or more, while the median of participants’ responses was used to obtain consensus on the activity standards.

**Results:**

The JDs of dietitians were a useful baseline for the consensus exercise as there were no other suitable source documents. The response rate was 100% for all three rounds of the Delphi survey. Dietitians reached agreement (consensus ≥ 70%) on 92% of proposed workload components and activity standards. Following the removal of duplicate and certain administrative activities, a total of 15 health, 15 support and 15 additional service activities with aligned activity standards resulted from the consensus exercise.

**Conclusion:**

The Delphi technique was a suitable method for reaching agreement on workload components and activity standards for dietitians at South African central and tertiary public hospitals. The findings from this study can now be used to compile a standardized list of workload components and activity standards and ultimately to determine dietetic staffing needs for the central and tertiary public hospital level of care.

## Background

Globally, there is a pressing need to invest in human resources for health (HRH) to address shortages and improve the distribution of health workers, ultimately ensuring universal health coverage [1]. South Africa, compared to other African countries, has a higher health worker density (> 4.45 per 1000 population) meeting the proposed Sustainable Development Goal index threshold [2]. However, the availability of health workers alone is insufficient; accessibility, acceptability, and quality of health workers are crucial factors to truly make a difference [1, 2]. Recognizing this need, the South African HRH strategy was developed through a ministerially appointed task team [3], with the aim of investing in the country’s workforce. While the strategy included health worker densities for several disciplines, it did not provide any data specifically for dietitians [3].

Dietitians are recognized as registered professionals responsible for providing community nutrition, therapeutic nutrition, and food service administration in South Africa, making them significant contributors to both preventative and curative services [4]. However, the absence of a national workforce planning tool to address nutrition workforce challenges can hinder the effective implementation of nutrition interventions and their desired outcomes [5]. The WHO, Workload Indicators of Staffing Need (WISN) tool has been successfully implemented for evidence-based workforce planning in several countries [3, 6–9]. The tool assists policymakers and managers in improving staffing equity across regions and facility types by developing workload components and activity standards tailored to specific disciplines [10–13]. While WISN has been implemented in South Africa previously to determine staffing needs in the primary health care setting, further research is needed to evaluate its suitability in the hospital setting [14].

The WHO WISN methodology consists of eight steps for determining workforce requirements. Steps 3 and 4 involve gathering key information required in the method, namely activities performed by a given cadre on a daily basis at a specific health service delivery level (i.e., workload components), and the time it takes a cadre to conduct these activities (i.e., activity standards), respectively. Therefore, defining of workload components (step 3) and setting of activity standards (step 4) based on the WISN methodology can assist in determining the actual work activities that take up most of a dietitian`s daily working time [6]. The development of these country, context and dietetic specific workload components and activity standards formed an essential component of the broader study whose aim is to develop a staffing norm framework for dietitians [6, 12]. Cadre-based expert working groups can be used to define these ‘two crucially important steps in the WISN method’ [6]. The Delphi technique, a method introduced in 1963 to obtain reliable opinion consensus of a group of experts through controlled questionnaires and feedback [15], was employed in this study. The online Delphi method was found to be the most feasible approach to gather input from head dietitians from 21 different hospitals and the nine provinces of South Africa, forming a cadre-based expert consensus group for this study [16, 17]. This technique has been widely used in various disciplines due to its cost-effectiveness and convenience when dealing with incomplete knowledge [17, 18]. Using a panel of experts, an online platform for questionnaire distribution, sequential questionnaires, and guaranteed anonymity for participant responses, the Delphi method enhances the rigor of studies and provides a quick and simple way to obtain data and guide group opinion towards consensus [16, 17, 19].

Currently, the professional scope of dietetics only provides a broad overview of the activities as related to dietetics in South Africa and does not provide a detailed guide for the specific daily work activities of dietitians [4]. Furthermore, the Health Professions Council of South Africa does not provide a scope of practice for dietitians. To determine the specific daily work activities of dietitians, we relied on the job descriptions (JDs) of dietitians as an appropriate source document. This helped establish an initial baseline and provided a focused framework for the Delphi consensus exercise [4, 17, 19]. The purpose of this paper is to describe the process followed in gathering expert opinions from dietitians and identifying consensus regarding the proposed dietetic WISN-based workload components and activity standards [6, 20] for central and tertiary public hospitals. Central and tertiary hospitals offer similar services; however, central hospitals serve a larger population. This study is a part of a larger study to determine the staffing needs of dietitians at this level of care.

## Methods

Participants were selected using non-probability purposive sampling methods to ensure appropriate representation [17, 18]. Data collection took place from February 2022 to May 2022. Data were collected in two phases.

In phase one, all nine provincial nutrition managers representing South Africa`s nine provincial health departments consented and participated in an online survey. The managers provided the JDs of dietitians working in their provinces. The JDs were then thematically analyzed using Microsoft Excel. The thematic analysis was done according to the three categories of the WISN workload components which formed the baseline for phase two of the study [6]. Health service activities were defined as “activities performed by all dietitians and for which annual statistics are regularly collected.” Support service activities were defined as “important activities that support health service activities, performed by all dietitians but for which annual statistics are not regularly collected.” Lastly, WISN defines additional activities as “activities performed only by certain members of the staff category and for which annual statistics are not regularly collected” [6].

Phase two included 21 out of a total 22 head dietitians at central and tertiary public hospitals who consented to participate in a survey using the online Delphi technique [21]. Although the ideal number of participants for a Delphi exercise is unspecified, approximately 10–15 participants may be sufficient for a homogenous sample [18–20]. By selecting the head dietitians of hospitals, we ensured that the participants were representative of dietitian work activities [16, 18]. Participants were given details about the study (purpose, their role as participants, the process, and expected outcomes) prior to commencement. This was done via email and participants were also offered the opportunity to have either telephonic or virtual meetings for further clarification if required [18]. Questionnaires in all rounds were presented as three parts: Part A: health service activities, Part B: support service activities, and Part C: additional activities together with aligned definitions to allow for a clear distinction between the three categories [6]. Participants were given the definition of activity standards “the time necessary for a well-trained, skilled and motivated dietitian to perform an activity to professional standards in the local circumstances” [6] and they were asked to provide the time required to perform each of the activities in the three workload component categories. Participants completed structured questionnaires including both closed and open-ended questions or practice statements during each of the three rounds until consensus was achieved [18]. Closed-ended questions or practice statements were presented using a 5-point Likert Scale (strongly disagree, disagree, do not disagree or agree, agree, and strongly agree). We piloted the first-round questionnaire to assess user-friendliness. Dietitians from regional, rather than central and tertiary public hospitals, participated in the pilot study to avoid possible contamination [17, 20, 21]. Participants were asked to evaluate the pilot questionnaire for clarity of concepts, phrasing of questions, relevance to the target group, length and time allowance, and overall user-friendliness.

The second and third questionnaires were based on the responses obtained from the first and second rounds, respectively [16]. Participants had two weeks to complete each round and were sent reminder emails before the deadline [18]. Participants requesting more time to complete the questionnaire were accommodated with reasonable extensions to avoid delays in subsequent rounds. Data were collected using Qualtrics, which allowed participants the flexibility and freedom to complete questionnaires without imposing on daily activities [18]. Following each round, we gave controlled feedback giving the participants an opportunity to revise their opinions with an informed knowledge of the views of the other participants [16, 18, 22]. Following the final round of the Delphi survey, we aggregated the data to identify the final workload components and activity standards [22]. Data were analyzed using Microsoft Excel and the Statistical Package of Social Sciences (SPSS). The agreement threshold for the Delphi technique depends on sample size, aims of the research, and resources available for the study [18]. In our study, we applied a 70% agreement threshold, as previously recommended, while considering the stability of responses between rounds [17, 18].

We also concurrently determined the activity standards of the aligned workload components as proposed by the participant group. Final activity standards were based on the median of the responses obtained [18]. The final lists were deemed as a standardization of WISN-based workload components and activity standards for central and tertiary public hospitals based on the expert opinion of head dietitians [6].

## Results

### Phase one: job descriptions

Of the 21 South African central and tertiary public hospitals, filled dietetic posts are as follows: one hospital employs a Deputy Director (DD), nine hospitals have filled Assistant Director (ASD) posts, 20 hospitals have dietitians employed as Chief Dietitians (CD), and 20 hospitals employ dietitians in Production (PD) posts (entry-level dietitian). None of the 21 hospitals had dietitians employed in all four dietetic ranks from January to April 2022. We obtained JDs for all ranks (DD, ASD, CD, PD) of dietitians. Sixteen hospitals provided JDs of CDs, 14 provided JDs of PDs, while nine and one provided JDs for ASD and DD, respectively. The JDs were categorized into either health service activities (Table 1), support service activities (Table 2) or additional service activities (Table 3) workload components as defined in the WISN user’s manual [6].

**Table 1:** Workload components and activity standards for health service activities

Heath service activities	Strongly agree (%)	Agree (%)	Do not agree/disagree (%)	Disagree/strongly disagree (%)	Activity standard (min per patient)
Delphi round one					
Ward rounds (individual and multidisciplinary)	95	5			10
Patient screening	71	14	15		5
Inpatient consultation and treatment (New)	100				30
Inpatient nutritional assessment (ABCDE) and diagnosis	100				15
Inpatient calculation of nutritional requirements and development of nutrition intervention plans	95	5			15
Inpatient nutrition support and dietary counselling	95	5			30
Inpatient consultation and treatment (follow-up)	95	5			15
Inpatient referral, communication with the multidisciplinary team and related activities	90	10			10
Outpatient consultation and treatment (new)	81	19			45
Outpatient nutritional assessment (ABCDE) and diagnosis	86	14			15
Outpatient nutritional plan and intervention including dietary counseling	86	14			30
Outpatient consultation treatment (follow-up)	67	28	5		30
Newly proposed health service activities: Delphi round two					
Outpatient consultation*	81	5	14		
Outpatient follow-up*	62	10	19	10	
Outpatient/specialist clinics (cerebral palsy, diabetes, etc.)	71	14	14		45
Report writing and patient notes	86	10	5		15
Referral process including writing of letters (between health facilities)	76	5	19		10

*Duplicate items were deleted from the final list of workload components

**Table 2:** Workload components and activity standards for support service activities

Support service activities	Strongly agree (%)	Agree (%)	Do not agree/disagree (%)	Disagree/strongly disagree (%)	Activity standard (h per year)
Delphi round one					
Administrative functions related to ordering of specialized diets and therapeutic nutrition (PN and EN) ^‡^	81	14		5	96
Monitor wastage and usage of PN and EN^‡^	76	19		5	48
Food service management (developing and updating of therapeutic diets and related diet sheets)	57	29	5	10	24
Monitor food service rendered by out-sourced company^†^	52	14	24	0	
Participation in journal reviews and working groups	76	10	14		38
Dietetics departmental meetings	86	14			38
Meetings with industry representatives and other stakeholders	52	43		5	18
Own performance development and management system (PMDS) reporting	90	10			8
CPD activities	81	19			24
Orientation of new staff*	76	24			24
In-service training to the multidisciplinary team and food service team*	71	24		5	12
Students mentoring (training), evaluation and reporting (Including meeting with universities)	71	24	5		90
Attend training (generic)	76	19	5		18
Recordkeeping and statistics	86	14			48
Peer reviews and clinical audits	81	14	5		24
Development and review of policies, protocols, and guidelines (Including related IEC materials)	76	19		5	36
Newly proposed health service activities: Delphi round two					
Outpatient health awareness events/campaigns/open days (planning and participation)	52	24	10	15	20
In-service training to the multidisciplinary team (nurses, doctors, etc.)	81	19			12
In-service training to the food service team	52	19	14	15	8
Dietetic administrative functions (telephone calls, emails, booking appointments, photocopying, etc.) ^‡^	48	29	10	15	191
Patient administration (patient handover, home care plans, recipes, etc.) ^‡^	57	24	19	0	153
Hospital committee/ Internal stakeholder meetings	52	43	5	0	44
Report writing (patient reports, medico-legal reports, etc.) *	43	29	24	5	38
The procurement process (ordering, receiving and monitoring of enteral feeds)*	67	14	10	10	137.5
Monitor and audit foodservices (In-house or outsourced as applicable) ^†^	48	14	19	20	

^*^excluded from the final list due to duplication; ^†^excluded due to low agreement, ^‡^excluded due to being administrative services that can be performed by other staff

**Table 3:** Workload components and activity standards for additional service activities

Additional service activities	Strongly agree (%)	Agree (%)	Do not agree/disagree (%)	Disagree/strongly disagree (%)	Activity standard (h per year)
Delphi round one					
Managerial duties (risk management, planning of duty rosters)	86	14			24
Financial management (budgeting and procurement) *	76	24			24
Asset management and physical resource management*	67	24	10		24
Develop departmental plans (strategic, business, and operational)*	71	29			16
Develop and review policies/strategies/guidelines/protocols and norms and standards*	76	24	0		24
Evaluate and monitor the implementation of policies/strategies/guidelines/protocols and norms and standards	71	24	5		24
Human resource management (grievances and disciplinary processes, HPCSA registration and compliance, attendance and leave register)	86	5	10		48
Recruitment, selection, and appointment of new staff*	81	19			16
Training, support and supervision of lower-level staff and community service dietitians	90	5	5		191
Performance development and management system (PMDS)	90	10			20
CPD activities*	81	19			24
Report writing, validations, and presentations	67	29		5	36
Participation in accreditation of facilities for student training*	48	38	14		8
National core standards (QIP)-develop plans, evaluation, and reports	62	33		5	19
Planning and coordination of departmental meetings	76	24			36
District, provincial INP and allied meetings	76	10	10	5	24
Participation in research activities	57	33		10	24
Food service management, development and costing of therapeutic diets (cycle menus, menu analyses, standardize recipes)*	52	29	10	10	16
Education, training, and supervision of foodservice/diet kitchen staff/milk kitchen/tube feed personnel	67	10	10	15	53
Ward rounds (foodservice)^†^	43	19	14	24	
Generate reports (meals, incidents, infection control)*	43	29	5	24	37
Operational management of human milk bank^†^	43	24	10	24	
Newly proposed health service activities: Delphi round two					
Outreach activities (Community or lower-level activities)†	14	19	33	35	
Hospital committee/ Internal stakeholder meetings*	48	43	5	5	44
MBFI mentor/committee participation and activities	43	33	19	5	24
Audits (stock take and stock take audits, diet sheet audits, equipment audits, etc.)	57	38	5	0	24
Stock takes of enteral feeds and supplements*	62	33	0	0	57
Develop and review departmental plans (Strategic, Business and Operational)	67	29	0	5	16
Asset management and physical resource management (including dietetic related equipment monitoring, repair, and monitoring) *	43	57	0	0	24

*excluded from the final list due to duplication; ^†^excluded due to low agreement

## Phase two: the Delphi process

### Round one

The 21 participants agreed on all 12 proposed health service activities (> 85%). Participants proposed five additional health service activities (Table 1). These newly proposed activities were summarized and carried forward into round two. Fifteen of 16 support service activities (Table 2) achieved agreement of 86 to 100%. One activity “monitor food services by an out-sourced company” scored 66%. Participants proposed an additional nine support service activities that were evaluated in round two (Table 2). Twenty of 22 additional service activities scored between 72 and 100%, meeting the consensus threshold. Two activities “ward rounds for food service” and “operational management of the human milk bank” scored 62% and 67%, respectively. Participants proposed seven new, additional service activities (Table 3). Activity standards were proposed for all agreed upon workload components in this round.

## Round two

The second questionnaire was based on the responses obtained in the first round [19]. Participants were provided with an anonymous summary of all responses from round one and given an opportunity to reflect and revise their opinions [19], [23]. At this stage, participants were requested to propose activity standards for all newly proposed workload components. In round two, participants rated the newly proposed health service (Table 1), support service (Table 2), and additional service activities (Table 3). The five newly proposed health service activities scored between 72 and 96% agreement. Eight of the nine newly proposed support service activities scored between 71 and 100% agreement with the one activity “monitor and audit foodservices—in house or outsourced as applicable” scoring 62%. Six of the seven newly proposed additional service activities scored between 91 and 100% with one activity “outreach activities” only scoring 33% agreement.

## Round three

We only re-visited activities that scored < 70%, representing diverging views of the participants. Participants were given another opportunity to review these activities and to justify or substantiate their scoring in this round [17, 19]. None of the health services activities scored less than 70%, so none if these activities were reviewed in round three. The support service activity “monitor and audit foodservices-in-house/outsourced as applicable” scored less than 70%. Three iterations later, the activity remained with a constant score of 62% showing little or no change in participant responses adding to the reliability of the responses [18]. Participants provided several reasons for either agreeing or disagreeing. Participants who agreed with the “monitor and audit foodservices-in-house/outsourced as applicable” activity, explained that *“I would be in agreement, provided that a food service dietitian is employed at the tertiary setting.”* Other participants stated that,

*“Outsourced food services should be monitored/audited at least once/quarter to monitor compliance to contractual stipulations. Food services have so many variables that need to be monitored, including compliance to menu's, production, portion sizes, financial parameters, ration scales, stock ordering, storage, shelf life of items, wastage of special diets, supplements - budgeting, costing, stock control.”*

*“Outsourced kitchens need to be supervised to ensure that patients are receiving what they are supposed to. To ensure patients are receiving quality meals. Monitor preparation and serving as well.”*

*“Monitoring and auditing of food services is essential as it has a direct impact on patient care. Current staffing however does not allow for this activity to the detriment of our patients.”*

Participants who disagreed explained the following:

*“Food service managers at provincial/national office should audit food services. Central hospitals have foodservice managers that do not report to dietitians.”*

*“Qualified foodservice managers need to be employed at this level as per NDOH FSM (National Department of Health Food Service Management) policy. Technical support meetings need to be held between dietetics and foodservice units to address challenges.”*

*“All institutions have FSU (Food Service Unit) managers (some also at AD level). It is their own responsibility to monitor and audit their service. Dietitians can be consulted regarding special diet requirements.”*

One additional service activity, “outreach activities-community or lower-level activities” did not reach the consensus threshold of 70% (Table 3). Participants who agreed with this activity stated:

*“Lower-level hospitals do not have dietitians and usually only have community service dietitians and usually require some support and supervision.”*

*“Dietitians need to visit clinics/ schools where no dietitians are allocated.”*

*“Outreach to lower-level hospitals and communities helps in supporting hospitals with guidelines on management of patients and when to refer patients to tertiary institution or next level of care. Also, to assist with resources that they might lack.”*

*“Outreach should be done with regards to training and workgroups and journal clubs. At a tertiary hospital there is a lot of expertise that can be shared through outreach.”*

Participants who disagreed with this activity stated the following:

*“Dietitians at tertiary hospitals should prioritize tertiary services, while the dietitians employed at district level should be responsible for outreach activities and other primary health services.”*

*“If the staff is adequate at a facility, then it is possible to do outreach. Outreach makes no sense if existing dietitians are not able to cover the entire service required at their own facility.”*

*“We do not do outreach to community as we have community-based dietitians working in the clinics.”*

All the agreed upon workload components and activity standards were reviewed in round three. A total of 71 workload components (17 health, 25 support, and 29 additional service activities) were proposed including those newly added by participants in the first two rounds. Following the consensus process, a total of 66 workload components (17 health, 23 support and 26 additional service activities) met the agreement threshold of 70% or more. This resulted in a 92% agreement rating on all proposed and newly added workload components.

The tables were further reviewed and verified by all the researchers to remove any duplicate activities, resulting in the final set of workload components and activity standards for dietitians at central and tertiary public hospitals. The final lists included several administrative activities performed by dietitians that may be performed by support staff. Such activities were also removed to allow for a more accurate representation of the activities that dietitians should be performing at central and tertiary public hospitals. The reviewed final lists following the removal of duplicate and certain administrative activities resulted in a total of 45 workload components (15 health, 15 support and 15 additional service activities) together with aligned activity standards (Tables 4, 5, 6).

**Table 4:** Final health service activities and related activity standards

Health service activities (≥ 70% agreement)	Activity standard based on the median (minutes per patient)
Ward rounds (individual and multidisciplinary)	10
Patient screening	5
In patient consultation and treatment (new)	30
In patient nutritional assessment (ABCDE) and diagnosis (new)	15
In patient calculation of nutritional requirements and development of nutrition intervention plans (new)	15
In patient nutrition support and dietary counselling (new)	30
In patient consultation and treatment (FU)	15
In patient referral, communication with the multidisciplinary team and related activities	10
Outpatient consultation and treatment (new)	45
Outpatient nutritional assessment (ABCDE) and diagnosis (new)	15
Outpatient nutritional plan and intervention including dietary counselling (new)	30
Outpatient consultation and treatment (FU)	30
Outpatient specialist clinics	45
Report writing and patient notes	15
Referral process between health facilities	10

**Table 5:** Final support service activities and related activity standards

Support activities (≥ 70% agreement)	Activity standard based on the median (hours per year)
Food service management (developing and updating of cycle menus, therapeutic diets and related diet sheets)	24
Development and review of policies, protocols, and guidelines (including related IEC materials)	36
Dietetics departmental meetings	38
Hospital committee/internal stakeholder meetings	44
Meetings with industry representatives and other external stakeholders	18
Own performance development and management system (PMDS) reporting	8
Continued professional development (CPD) activities	24
Participation in journal reviews and working groups	38
In-service training to the multidisciplinary team (nurses, doctors, etc.)	12
In-service training to the food service team	8
Student mentoring, evaluation and reporting (including meeting with universities and accreditation of facilities)	90
Attend training (generic)	18
Recordkeeping, statistics & report writing	48
Peer reviews and clinical audits	24
Outpatient health awareness events/campaigns/open days (planning and participation)	20

**Table 6:** Final additional service activities and related activity standards

Additional activities (≥ 70% agreement)	Activity standard based on the median (hours per year)
Managerial duties (risk management, financial management (budgeting and procurement), asset management, planning of duty rosters)	24
Audits (stock take and stock take audits, diet sheet audits, equipment audits, etc.)	24
Develop and review departmental plans (strategic, business and operational)	16
Evaluate and monitor the implementation of policies/strategies/guidelines/protocols and norms and standards	24
Report writing, validations and presentations	36
Human resource management (recruitment, selection of new staff, grievances and disciplinary processes, HPCSA registration and compliance, attendance and leave register)	48
Orientation of new staff, training, support and supervision of lower-level staff and community service dietitians	191
Performance development and management system (PMDS)	20
Participation in research activities	24
National core standards/quality improvement programs (QIP)-develop plans, evaluation, and reports	19
Planning and coordination of departmental meetings	36
District, provincial integrated nutrition program (INP) and allied meetings	24
Mother baby friendly initiative (MBFI) mentor/committee participation and activities	24
Education, training and supervision of foodservice/diet kitchen staff/milk kitchen/tube feed personnel	53

## Discussion

This study highlighted the strengths of using the online Delphi method together with a cadre-based expert working group to determine consensus on workload components and activity standards for dietitians in South Africa [6]. Using the online Delphi method, we were able to successfully obtain a 92% consensus rating on proposed workload components and activity standards. Only 8% of the activities fell short of the agreement threshold. We used JDs as the source document to provide a baseline while the iterative process created the ideal platform for further investigation, discussion, and agreement on the workload components and activity standards.

The online Delphi method allowed us to gather expert opinions and circumvented the need to meet physically while providing “real time and real-world data” in a short space of time [18, 19]. Compared to other WISN studies, the online approach limited the need for additional logistical arrangements, costs, and the need to remove health professionals from their work setting while allowing for a nationally representative sample [8, 10]. We ensured content validity by exploring the opinions of head dietitians with both an interest and expert knowledge on the workload components of dietitians [17, 18]. Quasi-anonymity ensured that the responses and opinions of participants remained strictly confidential and allowed participants the opportunity to share their views and opinions freely, limiting the risk of peer pressure and bias [16–22]. The concurrent rounds allowed for adequate consultation with experts to reach consensus on baseline activities. We also obtained a 100% response rate which added to the rigor and validity of our findings [18].

Two activities did not garner support from all participants. For the support services activity, “monitor and audit foodservices-in-household”, participants provided contrasting views. Some participants felt that dietitians should monitor and audit foodservices, while other participants felt that monitoring food services was the responsibility of the food services manager. In terms of additional activities, some participants felt that outreach activities should fall within the workload activities of dietitians, while other participants mentioned that outreach would only be possible if the dietitians were able to fulfill their existing duties. Dietitians at district hospitals might also be better suited to providing outreach services. For these activities, a large proportion of the group indicated a neutral response for both activities and making it difficult to delineate clear agreement or disagreement. Although these activities did not meet the 70% consensus threshold, we recommend that individual hospitals explore these two activities to determine their practical use.

## Limitations

This study had several limitations. The online platform introduced challenges with internet connectivity and limited access to computers for some participants; however, this was managed by conducting follow-up telephonic interviews. Different hospitals also provided different JDs, with some JDs providing detailed information on actual activities while others were very vague and only included broader key performance areas. We had to rely on the expertise of the researcher as a dietitian to extract activities as guided by the WISN definitions for the three categories of workload components [6]. These workload components thus served as a predefined set of activities for further exploration by the expert group [19], [23].

## Conclusion

The Delphi technique was a suitable method for obtaining consensus on WISN workload components and activity standards for dietitians at South African central and tertiary public hospitals. Although the JDs of dietitians were not standardized, we were able to identify a standard set of workload components and activity standards, which may allow for the possible standardization of JDs, providing a better representation of the actual activities performed by dietitians. The findings of this study can serve as a reference in future WISN studies that aim to assess the staffing requirements of dietitians at central and tertiary level public hospitals in South Africa.

## Data Availability

The datasets generated and/or analyzed during the current study are not publicly available due to these being the property of the South African Department of Health but are available from VNN on reasonable request and with permission and approval from the South African Department of Health and its corresponding research committees at provincial and hospital level.

## Abbreviations

ASD: Assistant Director
CD: Chief Director
DD: Deputy Director
HPCSA: Health Professions Council of South Africa
JDs: Job descriptions
PD: Production dietitian
SPSS: Statistical Package of the Social Sciences
WHO: World Health Organization
WISN: Workload Indicators for Staffing Need
